# Does the Site of Anterior Tracheal Puncture Affect the Success Rate of Retrograde Intubation? A Prospective, Manikin-Based Study

**DOI:** 10.1155/2013/354317

**Published:** 2013-06-26

**Authors:** Eric A. Harris, Kristopher L. Arheart, Kenneth E. Fischler

**Affiliations:** ^1^Department of Anesthesiology, Perioperative Medicine, and Pain Management, University of Miami/Miller School of Medicine, Miami, FL 33136, USA; ^2^Division of Biostatistics, Department of Epidemiology and Public Health, University of Miami/Miller School of Medicine, Miami, FL 33136, USA; ^3^Jackson Memorial Hospital, Miami, FL 33136, USA

## Abstract

*Background.* Retrograde intubation is useful for obtaining endotracheal access when direct laryngoscopy proves difficult. The technique is a practical option in the “cannot intubate / can ventilate” scenario. However, it is equally useful as an elective technique in awake patients with anticipated difficult airways. Many practitioners report difficulty successfully advancing the endotracheal tube due to anatomical obstructions and the acute angle of the anterograde guide. The purpose of this study was to test whether a more caudal tracheal puncture would increase the success rate.* Methods.* Twenty-four anesthesiology residents were randomly assigned to either a cricothyroid or a cricotracheal puncture group. Each was instructed how to perform the technique and then attempted it on a manikin at their assigned site. Data collection included whether the trachea was intubated, the number of attempts required, and the total time. *Results.* Both groups displayed a high degree of success. While the group assigned to the cricotracheal site required significantly more time to perform the procedure, they accomplished it in fewer attempts than the cricothyroid group. *Conclusion.* Retrograde intubation performed via a cricotracheal puncture site, while more time consuming, resulted in fewer attempts to advance the endotracheal tube and may reduce *in vivo* laryngeal trauma.

## 1. Introduction

Retrograde intubation of the trachea (RI) is an established airway management technique that can be used to place an endotracheal tube (ETT) when more conventional methods (e.g., direct laryngoscopy) have failed. The American Society of Anesthesiologists (ASA) difficult airway algorithm groups RI with other techniques (laryngeal mask airway (LMA), fiberoptic intubation, light wand, etc.) in the nonemergency limb of the pathway (i.e., cannot intubate but can ventilate the patient with either a mask or an LMA [1]). RI also has a role in the elective management of the difficult airway, where its chief strength is that it requires no visualization of the glottic structures. Hence, it can be performed in an airway that is soiled with blood or secretions—conditions that could make a fiberoptic intubation difficult or impossible [2]. It can be performed under general anesthesia with spontaneous or assisted ventilation but can also be performed under MAC or solely with the application of topical anesthesia to the airway. RI has been documented in pediatric patients as young as 5 months old [3]. The elective use of RI has been well documented in patients with angioedema [4], deep neck infections [5], burns [6], trismus [7], musculoskeletal disorders [8], and patients in the prehospital or emergency room [9] and trauma [10, 11] settings. Contraindications to the procedure include coagulation abnormalities, infection at the intended site of the puncture, pretracheal mass, and poorly palpable landmarks in the neck [12]. Adverse effects, albeit uncommon, include pneumomediastinum [13], infection, bleeding, and Macklin effect [14]—blunt traumatic alveolar rupture, air dissection along the bronchovascular sheaths, and spreading of this blunt pulmonary interstitial emphysema into the mediastinum. Sore throat is reported in 60% of patients after RI [15] to be slightly higher than the incidence noted after direct laryngoscopy [16–18].

The technique of RI is relatively simple to perform, consisting of a series of steps that are each individually familiar to most anesthesia providers [19] (cricothyroid puncture, topical application of local anesthesia to the upper airway, and advancement of an ETT over an introducer). Yet despite this, anecdotal evidence suggests that the procedure is not widely taught and felt by some to be an antiquated technique in a world of fiberoptic visualization tools. As a result, there may be a reluctance to consider this technique when confronted with an amenable situation [20]. While many clinicians have never tried to perform an RI, either due to lack of training or a misperception that the technique is excessively invasive, others have been disillusioned by initial attempts that resulted in esophageal intubations. They have discovered through trial and error that the anatomy of the laryngeal region is such that when the wire enters the trachea at the midpoint of the cricothyroid membrane, it is only approximately 9.8 mm distal to the vocal cords in an average sized adult [21] and as little as 5 mm in children [22]. As the ETT is advanced over the wire (or an anterograde guide that has been loaded over the wire), it can only be advanced to the point where the wire punctures the anterior tracheal wall—further advancement toward the carina can only be accomplished when the wire is withdrawn from the anterior neck. The short distance between the cricothyroid membrane and the vocal cords makes it easy for the unattached wire to inadvertently be withdrawn above the cords, thereby losing tracheal access and increasing the risk of a failed intubation.

The purpose of this prospective, manikin-based study was to test whether a more caudal site of anterior tracheal puncture, allowing more distance between the tip of the wire and the vocal cords, would result in a significantly higher success rate of endotracheal tube placement and therefore a better learning experience for residents unfamiliar with the technique.

## 2. Materials and Methods

IRB approval was granted by our institution to perform a prospective cadaveric-based study using anesthesiology residents as the target population. Initial statistical analysis indicated that the study would be adequately powered by using 24 residents; we therefore chose 8 from each year of training (CA-1, CA-2, and CA-3). Participants were made aware that the involvement in the study was voluntary, with no reward or repercussions given for the choice to participate or refrain, and written consent was obtained. Residents with prior experience performing three or more retrograde intubations were excluded to ensure the absence of any bias. One resident (CA-3) was excluded based upon the latter criterion and was replaced by a different CA-3 resident.

**Figure 1:**
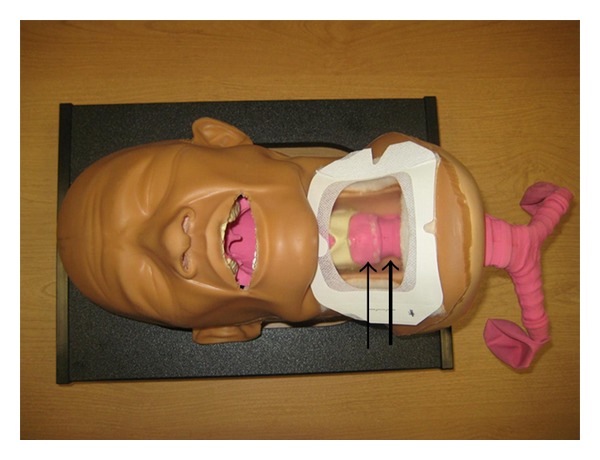
The thin arrow points to the cricothyroid membrane. The bold arrow points to the cricotracheal membrane.

**Figure 2:**
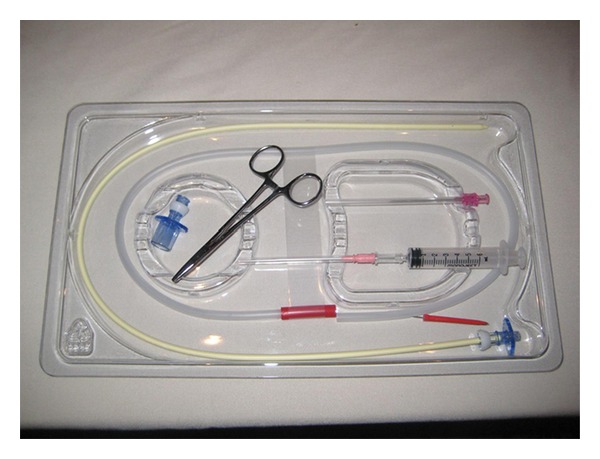
The Cook Retrograde Intubation Set (Cook Medical, Bloomington, IN, USA).

The cadavers chosen were preserved specimens provided by the University of Miami School of Medicine Department of Anatomy and Cellular Biology. After initial examination, it became apparent that preserved cadavers were unsuitable for the goals of the study. Anatomical rigidity made it difficult to advance an ETT over the anterograde guide, and it was impossible to determine if the ETT ultimately advanced into the trachea (extensive tissue sloughing within the trachea rendered fiberoptic examination useless). Due to ethical concerns about performing the study on freshly deceased patients, we decided to modify the protocol and use a manikin developed for teaching RI and cricothyroidotomy (Laerdal Medical, Wappingers Falls, NY, USA). Identification of the subcricoid membrane proved nearly impossible through the removable “neck” (a square of latex rubber over the cricothyroid region). This is in contrast to human patients, in whom the cricotracheal membrane is typically as easy to palpate as the cricothyroid membrane. To resolve this issue with the manikin, the removable latex neck piece was replaced with a clear large square Tegaderm (3 M Corporation, St. Paul, MN, USA) to allow better identification of the subcricoid region (Figure 1). RI was performed with a Cook Retrograde Intubation Set (Cook Medical, Bloomington, IN, USA, Figure 2) using a 7.5 ETT (Covidien-Nellcor, Boulder, CO, USA) that was lubricated externally with a water soluble gel.

Each resident was randomized to perform RI at either the cricothyroid or cricotracheal membrane such that each level of training had 4 residents attempting RI at each of the two sites. After a 1 : 1 tutorial with an anesthesiologist familiar with the procedure, each resident attempted to perform RI in the presence of only those involved in the study. The following parameters were recorded.

- Did the resident perform a successful RI? Success was defined as placing the ETT in the trachea with a maximum of 3 attempts at advancement over the anterograde wire. Intratracheal placement was confirmed by visual inflation of the lung balloons with Ambu bag ventilation via the ETT. Esophageal intubation was recorded as a failure to intubate with no further attempts allowed, as confirmation of an esophageal intubation required that the wire and anterograde catheter be removed from the manikin. Further attempts at RI would therefore require repeating the procedure from the beginning.
- How many attempts were required to advance the ETT over the anterograde guide?
- What was the time from puncture of the assigned membrane to confirmation of correct ETT placement?

## 3. Statistical Analysis

Success and success on first attempt were measured as binary variables (yes/no) and were reported as a percent. An exact chi-square analysis was used to compare the proportions between lower and upper sites because several of the cells had expected values less than five. An exact stratified chi-square analysis was also run to determine if stratification on year of training changed the results. Time in seconds to perform the procedure is reported as a least-squares mean and standard error. An analysis of variance was used to test for significant differences between the two sites. A second analysis of variance including year of training was run to determine if the inclusion of year changed the results. SAS 9.3 (SAS Institute, Inc., Cary, NC, USA) was used for all the analyses. Test results with a probability level (*P*) of 0.05 or less were deemed statistically significant.

## 4. Results

Table 1 reports the overall statistics for the comparisons of success rates, success on first attempt, and time to success between the lower and upper sites. Successful endotracheal intubation was accomplished in both groups at a very high rate. Ten of the 12 residents attempting RI via the cricothyroid membrane were successful (success rate 83.3%) while 11 out of 12 were successful via the cricotracheal membrane (success rate 91.7%). There was no significant difference in the proportion of success between the two groups (*P* = 1.000). The number of attempts needed to advance the tube past the laryngeal inlet (among those who successfully intubated the trachea) was significantly fewer in those using the cricotracheal membrane: the cricothyroid group averaged 1.8 attempts, with only 40% able to advance the tube on the first try. In contrast, every member of the cricotracheal group advanced the tube successfully on the first attempt (*P* = 0.004). There was no significant correlation between the number of attempts and the resident's level of training. The time to perform the procedure (among those that were successful) was significantly shorter for the group using the cricothyroid membrane (153.1 versus 188.7 seconds) (*P* = 0.044). Again, when the analyses were stratified by years of training, there was no change in the findings.

## 5. Discussion

First described in 1960 [23], the technique of RI has undergone numerous modifications throughout the years in an effort to make it more reliable and easier to perform. Significant improvements include the Water's technique, which uses an epidural catheter to guide the ETT [24], the use of the Murphy eye as a conduit for the retrograde wire [25], and the pairing of the retrograde technique with other airway management techniques in an effort to increase the success of placing the ETT in the trachea. Such adjunct techniques have included the use of a fiberoptic bronchoscope [26], a light wand [27, 28], a Combitube [29], a modified Eschmann stylet [30], an LMA [31, 32], a Mini-Trach II kit [33], a Cook airway exchange catheter [34], a gastric tube [35], and even a Fogarty embolectomy catheter [36]. Several clinicians have advocated the use of central venous access kits as a convenient collection of necessary supplies [37–39]. Perhaps the greatest enhancement to RI has been the introduction of the Cook Retrograde Intubation Set. The kit consolidates all of the components needed for the procedure, eliminating the need to scavenge supplies from a variety of sources. In addition to the 110 cm retrograde wire, the kit includes a 70 cm anterograde hollow guiding catheter which is inserted over the wire and advanced until contact is made with the anterior tracheal wall. Removal of the wire allows for advancement of the catheter deeper into the trachea, ostensibly increasing the chance of successfully placing the ETT in its desired destination. Furthermore, the anterograde guide is equipped with a pair of Rapi-Fit adapters, one with a standard 15 mm diameter and the other suitable for jet ventilation. This allows for the oxygenation of the patient who may require assistance prior to the placement of the ETT.

Despite advancements in the technique of RI, the threat of the possible dislodgement of the ETT or anterograde catheter after the release of the retrograde wire guide at the cricothyroid membrane [40] remains, resulting in an unsuccessful tracheal intubation [10, 41]. Anatomically, this is due to the proximity of the vestibular folds, laryngeal sinus, and the vocal cords to the inferior border of the thyroid cartilage [42]. The confluence of these structures in this small space, as well as the acute angle the ETT needs to navigate, make it likely that the ETT will catch against the anterior surface of the larynx as it is advanced. While some authors recommend a greater degree of tension be held on the retrograde wire to increase the odds of ETT passage [8], this tension may result in bleeding, edema, and trauma to the local tissues. Others recommend rotating the tube as it approaches the cords [43], but this evidence is anecdotal at best. A puncture site caudal to the cricothyroid membrane would seem to offer the advantage of a greater distance between the retrograde wire and the impinging structures, as well as a reduction in the acuity of the angle the ETT must negotiate into the trachea. The cricotracheal membrane, the first membrane caudal to the cricothyroid, seems an ideal candidate. It is typically as easy to identify *in vivo* as its more cephalad counterpart, despite its smaller vertical span (10 mm versus 6 mm). It is devoid of blood vessels or nerves, and sits cephalad of the thyroid gland (which typically ascends to the second tracheal ring, although a pyramidal lobe may extend toward the hyoid bone [44]). The distance from the vocal cords (2.5 cm) is double the distance between the cords and the standard cricothyroid puncture site, thereby lessening the chance that bleeding or edema at the puncture site will result in hoarseness or subglottic edema [42], as well as reducing the chance of directly injuring the vocal cords when the needle is introduced [45]. Other authors have postulated that the cricotracheal membrane may be a more suitable site for RI [42, 45], but, to our knowledge, no published studies comparing the different techniques exist.

We were surprised to see the high success rate of proper endotracheal tube placement using the cricothyroid membrane (83.3%); our clinical experience is more in the range of 70%. While the success rate between the two puncture sites was statistically insignificant, the cricotracheal puncture resulted in a significant reduction in the number of times that the ETT had to be advanced over the anterograde catheter until it entered the trachea. Clinically this is important, as it seems logical that continued attempts of advancement of the ETT against resistance would be a major cause of bleeding and edema. From a training perspective, repeated failure to advance the tube is likely to discourage the novice practitioner and prejudice him/her against this technique. The other surprising finding was that successful intubation using the cricotracheal membrane took significantly more time than using the cricothyroid membrane, even taking into account the fact that the cricothyroid site often required multiple attempts to advance the ETT. While this added time may seem like a clinical detriment in the difficult airway situation, closer examination of the situation reveals that it is not as troublesome as it seems. Referring back to the ASA difficult airway algorithm, the RI technique is recommended in cases where there is difficulty intubating the patient, but ventilation is satisfactory. (It is also indicated in an anticipated difficult airway in which the patient has not been induced.) Cases that fall on the “cannot intubate/can ventilate” limb of the pathway do not have the same degree of urgency as failed airways in which the patient cannot be intubated or ventilated. Thus the extra time taken by the participants in the cricotracheal group would occur concurrently with ventilation of the patient.

Initially, we considered performing the study as a “cross-over” study by having participants from each group perform the technique at both sites. However, we concluded that this might alter our results by introducing a bias to the second attempt. As we were seeking inexperienced practitioners, we thought that the second attempt would likely be improved by the participant having already performed the procedure once. Clinically, we assume that residents would have a single attempt to attempt this procedure. We therefore attempted to duplicate this scenario with the manikins, giving each resident a single attempt in an effort to simulate their *in vivo* success rates.

Our original protocol involved the use of preserved human cadavers instead of manikins. While other authors have successfully used preserved human cadavers to practice or teach RI [46, 47], we did not share the same success. First, the inherent stiffness of the cadavers made it difficult to negotiate the necessary angles when inserting the anterograde guide. More significantly, once the ETT was advanced, we found it difficult to confirm proper placement within the trachea. Fiberoptic examination revealed a large amount of soft tissue sloughing, which obscured any anatomy that would allow us to identify ETT position. Our next option was to use freshly deceased cadavers. However, this proposition was rejected by our IRB, which cited ethical concerns about approaching the families for consent. We therefore chose to use manikins that were specifically designed for percutaneous cricothyrotomy and RI. Other authors have also reported the successful use of manikins to simulate RI in humans [9, 35]. The manikin was designed for identification of the cricothyroid space; the cricotracheal area, however, was not easily palpable. We therefore chose to replace the removable opaque latex “neck” with a clear adhesive dressing to allow identification of both spaces.

While retrograde intubation may never ascend to the popularity of other airway management devices, it still presents a viable alternative for the management of the anatomically challenging airway in which adequate ventilation (either spontaneous or assisted) is ensured. As such, we feel it should be included in any thorough anesthesiology curriculum. Its limited teaching and subsequent scarce use are likely due to two separate factors: the misperceived, exaggerated invasive nature of the procedure [19] and the fact that the proximity of the cricothyroid puncture site to the vocal cords leaves a little room for error [48]. Further study using human patients is warranted to see whether the cricotracheal puncture site may result in a higher degree of accurate ETT positioning among novice practitioners, who may therefore be more likely to incorporate retrograde intubation into their practices.

## 6. Summary

This is a prospective, manikin-based study to determine if a cricotracheal membrane puncture (as opposed to the more traditional cricothyroid membrane puncture) results in a better rate of successful endotracheal intubation for residents inexperienced in performing retrograde intubation.

## Figures and Tables

**Table 1:** Site differences for success, first attempt, and time to perform the procedure.

	Cricotracheal	Cricothyroid	*P *	Stratified
Overall (*n* = 24)	(*n* = 12)	(*n* = 12)		
Success (%)	92	83	1.000	0.544
First attempt (%)	100	40	0.004	0.005
Time in seconds (mean ± se)	180 ± 9	153 ± 9	0.044	0.031
